# The Clinical Significance of Fluctuations in the Minute-to-minute Urine Flow Rate and in its Minute-to-minute Variability During Septic Events in Critically Ill Patients

**DOI:** 10.2478/rjaic-2020-0013

**Published:** 2020-12-31

**Authors:** Anna Shalman, Yoram Klein, Ronen Toledano, Yuval Wolecki, Yoav Bichovsky, Leonid Koyfman, Anton Osyntsov, Asaf Acker, Moti Klein, Evgeni Brotfain

**Affiliations:** 1 Department of Anesthesiology and Critical Care, General Intensive Care Unit, Soroka Medical Center, Ben-Gurion University of the Negev, Beer Sheva, Israel; 2 Division of Trauma, Sheba Medical Center, Ramat Gan, Israel; 3 Department of Orthopedic Surgery, Rabin Medical Center, Beilinson Campus, Petah Tikva, Israel; 4 Department of General Surgery B, Soroka Medical Center, Ben-Gurion University of the Negev, Beer Sheva, Israel; 5 Department of Orthopedic Surgery, Soroka University Medical Center, Ben-Gurion University of the Negev, Beer Sheva, Israel

**Keywords:** Urine flow rate, urine flow rate variability, sepsis, critically ill patients, intensive care unit

## Abstract

**Background:**

Septic events complicated by hemodynamic instability can lead to decreased organ perfusion, multiple organ failure, and even death. Acute renal failure is a common complication of sepsis, affecting up to 50-70 % of cases, and it is routinely diagnosed by close monitoring of urine output. We postulated that analysis of the minute-to-minute changes in the urine flow rate (UFR) and also of the changes in its minute-to-minute variability might lead to earlier diagnosis of renal failure. We accordingly analyzed the clinical significance of these two parameters in a group of critically ill patients suffering from new septic events.

**Methods:**

The study was retrospective and observational. Demographic and clinical data were extracted from the hospital records of 50 critically ill patients who were admitted to a general intensive care unit (ICU) and developed a new septic event characterized by fever with leukocytosis or leukopenia. On admission to the ICU, a Foley catheter was inserted into the urinary bladder of each patient. The catheter was then connected to an electronic urinometer – a collecting and measurement system that employs an optical drop detector to measure urine flow. Urine flow rate variability (UFRV) was defined as the change in UFR from minute to minute.

**Results:**

Both the minute-to-minute UFR and the minute-to-minute UFRV decreased significantly immediately after each new septic episode, and they remained low until fluid resuscitation was begun (*p* < 0.001 for both parameters). Statistical analysis by the Pearson method demonstrated a strong direct correlation between the decrease in UFR and the decrease in the systemic mean arterial pressure (MAP) (*R* = 0.03, *p* = 0.003) and between the decrease in UFRV and the decrease in the MAP (*R* = 0.03, *p* = 0.004). Additionally, both the UFR and the UFRV demonstrated good responses to fluid administration prior to improvement in the MAP.

**Conclusion:**

We consider that minute-to-minute changes in UFR and UFRV could potentially serve as early and sensitive signals of clinical deterioration during new septic events in critically ill patients. We also suggest that these parameters might be able to identify the optimal endpoint for the administration of fluid resuscitative measures in such patients.

## Introduction

Septic events complicated by hemodynamic instability can lead to decreased organ perfusion, multiple organ failure, and even death. Acute renal failure is a common complication of sepsis, affecting up to 50–70% of cases,[1,2] and it is associated with high mortality.[2] Bagshaw et al.[3] reported that acute kidney injury (AKI) significantly increased the intensive care unit (ICU) and in-hospital mortality of patients with sepsis and that this higher mortality was observed across all the severity categories of AKI. The pathophysiology of sepsis-induced AKI is multifactorial, involving the effects of any or all of global renal ischemia, cellular damage, and acute tubular necrosis (ATN), and it is relevant to note that AKI can develop even in the absence of hypoperfusion.[2] The risk of developing AKI after sepsis is particularly high in the elderly, in males, and in patients with severe illness, low urinary output, high central venous filling pressure, substantial vasopressor requirements, and/or a history of prior treatment with angiotensin-converting enzyme inhibitors or angiotensin II receptor blockers.[4]

It is essential to identify AKI early in septic patients because therapeutic and supportive maneuvers in these patients, such as vasopressor therapy administered without adequate fluid resuscitation, or the administration of potentially nephrotoxic

antibiotics (e.g., vancomycin or aminoglycosides) can aggravate sepsis-induced renal injury5. Current methods that are currently used to confirm the diagnosis of AKI and to assess its prognosis include urinalysis by the dipstick technique and estimation of the fractional excretion of sodium and urea5. These methods are commonly employed to help differentiate pre-renal AKI from ATN, but they remain insensitive and non-specific tools for gathering reliable information relevant to the early diagnosis of AKI. [5] In general, assessment of urine chemistry in critically ill patients, especially in cases of sepsis, is often unreliable due to fluctuations in the clinical course and severity of the septic process in each individual patient; the heterogeneity of kidney disease; and other confounding factors such as timing, pre-existing chronic kidney disease (CKD), and the administration of fluids, vasopressors, or diuretics.[4,5] Furthermore, a diagnosis of AKI inferred from elevation of the serum creatinine level or from the presence of oliguria is often made well after the window of opportunity for therapeutic or preventative intervention has passed.[5, 6, 7] Therefore, a system that provides close and continuous monitoring of urine output has the potential to be a crucial diagnostic tool in following the course of septic critically ill patients. The aim of this study was to analyze the diagnostic and prognostic significance of minute-to-minute changes in the urine flow rate (UFR) and in the urine flow rate variability (UFRV) in a group of critically ill patients who were admitted to a general ICU after major abdominal surgery and who subsequently developed a new septic episode during their ICU stay.

## Patients and methods

Soroka Medical Center is a 1000-bed tertiary-care university teaching hospital located in Beer Sheva in southern Israel. The study was retrospective and observational. We retrospectively collected the clinical and laboratory data of postoperative critically ill patients who developed a new septic episode while hospitalized in the general ICU at Soroka between January 2018 and January 2019. The clinical data were extracted from a computerized clinical information system (MetaVision®, iMDsoft®, Israel). The Human Research and Ethics Committee at Soroka Medical Center in Beer Sheva, Israel, approved the study (RN-0158-14-SOR).

### Inclusion criteria

Patients above the age of 18 were eligible for inclusion in the study if they were admitted to the ICU for observation following major abdominal surgery while afebrile and in a hemodynamically stable condition (as evidenced by the presence of a systolic blood pressure [SBP] of >90 mmHg and a mean arterial pressure [MAP] of >65 mmHg without vasopressor treatment) and subsequently during their stay in the ICU developed a new septic event, manifesting as fever accompanied by leukocytosis or leukopenia.

### Exclusion criteria

The following patients were excluded from the study: patients aged <18; patients with a history of chronic renal failure or CKD; patients who were hospitalized in the ICU for less than 72 hours; and patients whose medical records contained insufficient data.

### Variables and measures

Data were collected from the hospital’s electronic recording system and its laboratory database. We documented the patients’ demographic data (age, gender, weight); their vital signs (minute-to-minute UFR, urine output per hour, total fluid balance per hour, heart rate, arterial blood pressure, and body temperature); their laboratory parameters (hemoglobin level; arterial lactate and bicarbonate levels and pH; and serum urea, creatinine, and electrolyte levels); their primary diagnoses on admission to the ICU; their Acute Physiology and Chronic Health Evaluation II (APACHE II) scores on ICU admission; and the therapeutic measures that were undertaken during the9 hours following the patients’ admission to the ICU in regard to the administration of intravenous fluids.

### Urine flow monitoring

On admission to the ICU, a Foley catheter was inserted into the urinary bladder of each study patient. The catheter was then connected to a URINFO 2000™ device (FlowSense Medical, Misgav, Israel) – a collecting and measurement system that employs an optical drop detector to quantify urine flow through a measuring chamber. The detector enables the reliable calculation of UFRs at varying flow rates and osmolarities. The UFRV was defined as the change in UFR from minute to minute.

### Definition of a new septic event

A new septic event was defined as at least one documented increase in core temperature to above 38.5°C associated with either leukocytosis (white blood cell [WBC] count of >9000 cells/mL) or leukopenia (WBC count of <4000 cells/mL). All vital signs recorded during the 3 hours preceding the new septic event and during the 9 hours following the event were collected and analyzed.

### Scores

The APACHE II score was used to assess the severity of illness in each of the study patients.

### Statistical analysis

Parametric analysis was the preferred method of analysis for continuous variables. The parametric assumptions were assessed using a normal probability plot or the Shapiro– Wilk test for verification of normality and Levene’s test for verification of homogeneity of variances. The *t*-test was used accordingly. Categorical variables were tested using Pearson’s χ2 test for contingency tables. For the comparison of minute-to-minute UFRV, the coefficient of variation was calculated and analyzed by the Student’s *t*-test. The Pearson correlation coefficient was used to evaluate the linear relationship between the different continuous variables. To evaluate the possible non-linear association between the UFRV and the SBP or the MAP, we used local regression (LOESS) curves. A two-tailed *p* value of <0.05 was considered to be significant. All analyses were performed using SPSS version 20 software (SPSS, Chicago, Illinois).

## Results

We initially analyzed the clinical and laboratory data of 200 critically ill patients who were admitted to the ICU following major abdominal surgery. Of this group, 50 patients were eventually included in the study on the basis of the inclusion criteria. The demographic data of these patients and their APACHE II scores on admission are shown in Table 1.

**Table 1:** Demographic data and APACHE II^a^ scores on admission.

Septic critically ill patients (*n* = 50)	58.80 ± 19.74
Age, years (mean **±** SD)	58.80 ± 19.74
Gender (male)	31 (61%)
Weight, kg (mean **±** SD)	84.19 ± 22.64
APACHE II score (units, mean **±** SD)	21.68 ± 4.9

a Acute Physiology and Chronic Health Evaluation II.

**Figure 1:**
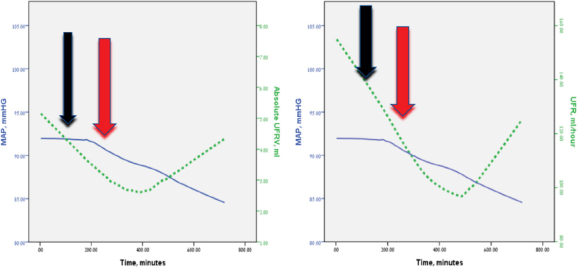
Correlation of urine flow rate variability (UFRV) (A) and urine flow rate (UFR) (B) with mean arterial pressure (MAP) at the time of new septic events (black arrows) and following initial fluid resuscitation (red arrows). Note that the UFRV and the UFR decreased progressively in parallel with the falling MAP and rose again after the administration of fluids

A sustained decline in the MAP and a slight increase in heart rate were observed prior to the septic event and were noted to persist for an average of 9 hours following the event; however, these changes were not found to be statistically significant (see Tab. 2 and Fig. 1). The minute-to-minute UFR and the minute-to-minute UFRV both decreased significantly before and after the septic event (*p* < 0.001 for both parameters; Tab. 2). Notably, the UFR and the UFRV decreased progressively in parallel with the falling MAP and then rose again after the administration of fluids (see Fig. 1).

**Table 2:** **Laboratory and clinical data of the 50 study group patients (mean ± SD)**.

	TIME1^**^	TIME2^**^	*p* value^*^
Mean arterial blood pressure (mmHg)	94.53 ± 14.84	88.21 ± 13.62	0.06
Heart rate (beat/min)	105.48 ±19.38	108.05 ± 20.50	0.06
Urine output per hour (mL)	147.56 ± 28.1	87.53 ± 15.8	<0.001
Minute-to-minute urine flow rate^a^ (mL/hour)	146.65 ± 117.30	106.65±72.0	<0.001
Urine flow rate variability (mean, mL)	5.18 ± 9.40	2.63 ± 3.35	<0.001
Hemoglobin (g\dL)	9.43 ± 1.16	9.03 ± 0.12	0.2
White blood cell count (k/ mm^3^)	9.9 ± 3.4	15.9±1.45	0.01
Bicarbonate arterial blood (mEq/L)	26.3 ± 4.35	20.3 ± 1.35	0.03
pH arterial blood	7.41 ± 0.01	7.31 ± 0.03	0.06
Arterial blood lactate (mmol/L)	1.19 ± 0.70	2.5 ± 1.4	0.04
Urea (mg/dL)	54 ± 15.5	74 ± 7.2	0.06
Creatinine (mg/dL)	0.8 ± 0.03	0.9 ± 0.1	0.08
Body temperature (°C)	36.08 ± 0.05	38.98 ± 0.05	0.03

** TIME1 – vital signs during the 3 hours following admission to the ICU; TIME2 – vital signs 9 hours after the septic event.

* A *p* value of less than 0.05 is considered to be statistically significant.

a Minute-to-minute urine flow rate is denoted as mL/hour urine volume as measured every minute by the URINFO 2000™ urinometer.

The blood arterial bicarbonate level was significantly decreased at 9 hours after the septic event (*p* = 0.03; Tab. 2), and the blood arterial lactate level, the WBC count, and the body temperature all rose significantly after the septic event (*p* = 0.04, 0.01, and 0.03, respectively; Tab. 2). There were no significant changes in the blood hemoglobin level (Tab. 2).

Statistical analysis by the Pearson method demonstrated a strong direct correlation between the decrease in the UFRV and the decrease in the MAP (*R* = 0.03, *p* = 0.004) and between the decrease in the UFRV and the decreasing urine output per hour (*R* = 0.031, *p* = 0.003). No correlation with heart rate was found. The correlation between changes in the UFRV and the UFR and changes in the mean arterial blood pressures is demonstrated in Figure 1.

## Discussion

Sepsis is a systemic disorder that can affect all organs of the body. At the microcirculatory level, it is characterized by leakiness of the capillary membranes, resulting in massive loss of intravascular proteins and plasma fluids into the extravascular space. In addition, diffuse vasodilation throughout the microcirculation alters capillary blood flow, leading to poor tissue perfusion and ultimately to septic shock – a form of distributive shock in which recovery of blood pressure cannot be achieved solely by the administration of additional intravenous fluids but requires concurrent administration of a vasoconstrictive agent such as noradrenaline and/or vasopressin.[8, 9, 10, 11]

Renal dysfunction that progresses to frank renal failure is a major cause of sepsis-induced morbidity and mortality.[8, 9, 10, 11] Although the exact mechanisms responsible for the development of renal failure in sepsis are unknown, its pathophysiology seems to involve complex and subtle mechanisms of cytokine and immune mediated microvascular and tubular dysfunction. Oliguria is frequent in ICU patients with sepsis-induced AKI. It appears to be a more sensitive marker of AKI than an increase in the serum creatinine level, and it has also been shown to be an independent risk factor for death.[12]

Restoration of tissue perfusion and optimization of hemodynamic status by fluid therapy and vasopressor infusion are the main goals of supportive therapy in sepsis and septic shock.[13] Furthermore, most reports have confirmed that the prevention of tissue hypoperfusion by targeted hemodynamic resuscitation before the onset of significant hypovolemia is associated with improved outcomes.[6] Because of the loss of intravascular volume in sepsis due to leaky capillary membranes and vasodilation, patients typically require a large amount of volume resuscitation to replace these losses, and clinicians can reduce the incidence of severe renal failure in septic patients by aggressive and appropriate volume resuscitation. However, the large volume of crystalloids given to maintain central blood pressure in the presence of endothelial injury frequently leads to pulmonary edema.[7, 8, 9] Therefore, a parameter that provides an early indication of hemodynamic recovery to enable fluid administration to be reduced or ceased in a timely manner would be extremely useful.

We have previously demonstrated[14,15] that continuous minute-to-minute UFR and UFRV measurements are efficient monitoring parameters in critically ill multiple trauma patients and that a decrease in minute-to-minute UFR and UFRV is predictive of hemodynamic deterioration in unstable multiple trauma patients.[14,15] In hypovolemic multiple trauma patients,[14] minute-to-minute UFR and urine flow variability decreased during the first 6 hours of ICU admission in parallel with decreases in SBP and MAP and increases in heart rate and arterial blood lactate levels. Importantly, multiple trauma patients in that study[14] had an “adequate” (about 1 cc per kilogram per hour) urine output. Thus, the minute-to-minute UFR/urine flow variability as a continuous, sensitive measurement has a significant clinical advantage and is superior to other vital parameters as an early diagnosis of hypovolemia in multiple trauma patients. Moreover, in a subsequent study[15] of multiple trauma patients who were normotensive on admission to the ICU and then developed hypotension within the next few hours, we found that a significant dynamic decrease in minute UFR and in UFRV amplitude preceded the overt decline in SBP and MAP that accompanied the patients’ progressive hemodynamic compromise. Furthermore, we showed that the **disappearance of UFRV amplitude (the flattened segment of the UFRV graphs depicted in Figs. 1A and 1B)** preceded the clinically evident drops in systolic and mean arterial blood pressures (SBP < 90, MAP < 60) by at least 30 minutes.[15]

In the present study, we investigated a group of critically ill patients who were admitted to an ICU for observation following major abdominal surgery and who developed a new septic episode during their ICU stay. We demonstrated that both the minute-to-minute UFR and the minute-to-minute UFRV decreased significantly before and after septic events and that the decrease in the UFRV correlated with decreases in both the MAP and the hourly urine output. Moreover, we found not only that the minute-to-minute UFR and UFRV decreased progressively in parallel with the falling MAP but also that these two parameters rose again after the administration of fluids (Fig. 1). We believe that in critically ill patients, dynamic changes in the minute-to-minute UFR and UFRV are extremely sensitive indicators of decreasing renal function and thus could potentially serve as signals of clinical deterioration during new septic events earlier than standard parameters such as blood pressure, heart rate, and hourly urine output. We also suggest that measurement of minute-to-minute UFR and UFRV might be able to identify the optimal endpoint of fluid resuscitative measures in these patients.

Our study has a number of limitations, the main ones being its retrospective design and the small number of patients who were surveyed. Because of the retrospective nature of the study, we could not assess the extent to which the minute-to-minute UFR and UFRV were influenced by active therapeutic interventions undertaken to stabilize the systemic blood pressure. Furthermore, at the time of the study, no other advanced hemodynamic monitoring systems (e.g., the Pulse Contour Cardiac Output system) were available to us for more precise assessment of the patients.

## Conclusion

We found that changes in the minute-to-minute UFR and UFRV correlate strongly with changes in the MAP in critically ill patients during new septic events. We feel that in critically ill patients, these parameters could potentially serve as early indicators of hemodynamic deterioration due to septic events and might also be able to identify the optimal endpoint of fluid resuscitative measures. In view of our findings, we suggest that a prospective randomized and interventional study should be undertaken to evaluate the potential role of measurements of minute-to-minute UFR and UFRV in improving the outcome of critically ill patients with sepsis.
